# Morphological changes and functional circRNAs screening of rabbit skeletal muscle development

**DOI:** 10.1186/s12864-021-07706-y

**Published:** 2021-06-24

**Authors:** Qi Zheng, Cuiyun Zhu, Jing Jing, Yinghui Ling, Shuaiqi Qin, Jiao Wang, Lisha Zha, Ya Liu, Fugui Fang

**Affiliations:** 1 College of Animal Science and Technology, Anhui Agricultural University, Anhui Hefei, P.R. China; 2 Anhui Province Key Laboratory of Local Livestock and Poultry Genetic Resource Conservation and Bio-Breeding, Anhui Agricultural University, Hefei, P.R. China

**Keywords:** Rabbit, Skeletal muscle, Morphology, CircRNA, RNA-seq

## Abstract

**Background:**

The temporal expression pattern of circular RNAs (circRNAs) across developmental stages is essential for skeletal muscle growth and functional analysis. However, there are few analyses on the potential functions of circRNAs in rabbit skeletal muscle development.

**Results:**

Initially, the paraffin sections showed extremely significant differences in the diameter, number, area and density of skeletal muscle fibers of the fetus, child, adult rabbit hind legs (*P* < 0.01). Then, RNA-seq libraries of these three stages were constructed. A total of 481 differentially expressed circRNAs (DE-circRNAs) and 5,658 differentially expressed genes (DEGs) were identified. Subsequently, DE-circRNAs, whose host genes were DEGs or non-DEGs, were analyzed by GO respectively. In the fetus vs. child group, up-regulated DE-circRNAs (whose host genes were DEGs) were related to muscle fiber structure, and down-regulated ones were related to mitosis. The up-regulated DE-circRNAs (whose host genes were non-DEGs) were involved in enzyme activity, methylation and glycosylation, and the down-regulated ones were involved in mitosis and catabolism. In the fetus vs. adult group, the up-regulated DE-circRNAs (whose host genes were DEGs) were related to skeletal muscle basic structure, and the down-regulated ones were also associated with cell proliferation. But the up-regulated DE-circRNAs (whose host genes were non-DEGs) were connected with regulation of histone ubiquitination, chromatin and organelles. The down-regulated DE-circRNAs were connected with the catabolism processes. In addition, novel_circ_0022663 and novel_circ_0005489, which might have coding potential, and novel_circ_0004210 and novel_circ_0001669, which might have miRNA sponge capability, were screened out.

**Conclusions:**

In this study, hind leg muscles of fetus, child and adult rabbits were collected for paraffin section and RNA-seq to observe the structural changes of skeletal muscle and obtain circRNA expression profiles at different stages. These data provided a catalog of circRNAs related to muscle development in New Zealand rabbits, allowing us to better understand the functional transitions in mammalian muscle development.

**Supplementary Information:**

The online version contains supplementary material available at 10.1186/s12864-021-07706-y.

## Introduction

The rabbit (*Oryctolagus cuniculus*) is regarded as an ideal meat-producing animal in theory, because of its short life cycle, short gestation period, large number of litters, and high feed conversion ability [1]. At the same time, the high protein, low cholesterol and fat characteristics of rabbit meat make it popular among consumers [2]. Leg muscle, the most economical part of meat rabbits, are the focus of molecular breeding. With the development and application of molecular biology and bioinformatics, it is possible to reveal the basis of meat rabbit production performance from the molecular level [3]. Therefore, finding genetic changes linked to meat quality traits has become particularly significant.

Circular RNA (circRNA) is a kind of non-coding RNA formed by covalently closing continuous loops [4]. Due to the circular structure, circRNAs have high stability and conservation in mammalian [5]. Recently, circRNAs are considered to have the ability to sponge miRNA, encode polypeptides or directly regulate transcription factors, thereby regulating a variety of processes comprised muscle development [6, 7]. For example, silencing of circSamd4 enhance the association of PUR protein with myosin heavy chain (MHC) promoter, thus inhibiting MHC to promote myogenesis [8]. circ-ZNF609 encode a polypeptide to specifically control the proliferation of myoblasts [9]. Moreover, circ-calm4 serve as an miR-337-3p sponge, and regulated the proliferation of pulmonary artery smooth muscle cells at the molecular level through the circ-calm4 / miR-337-3p / Myo10 signal transduction axis [10]. These studies use advanced techniques to enable molecules that were once unobservable to be identified.

The postpartum growth of skeletal muscle is mainly achieved by increasing the length and circumference of muscle fibers, not by increasing the number of muscle fibers. Therefore, this study selected fetus, child and adult rabbits’ muscle of hind legs to explore the circRNAs expression profiles at different stages and screened circRNAs that might have potential effects. Research related data provided novel clues for rabbit muscle development and molecular breeding.

## Result

### The morphological changes of rabbits’ skeletal muscle at different stages

**Fig. 1:**
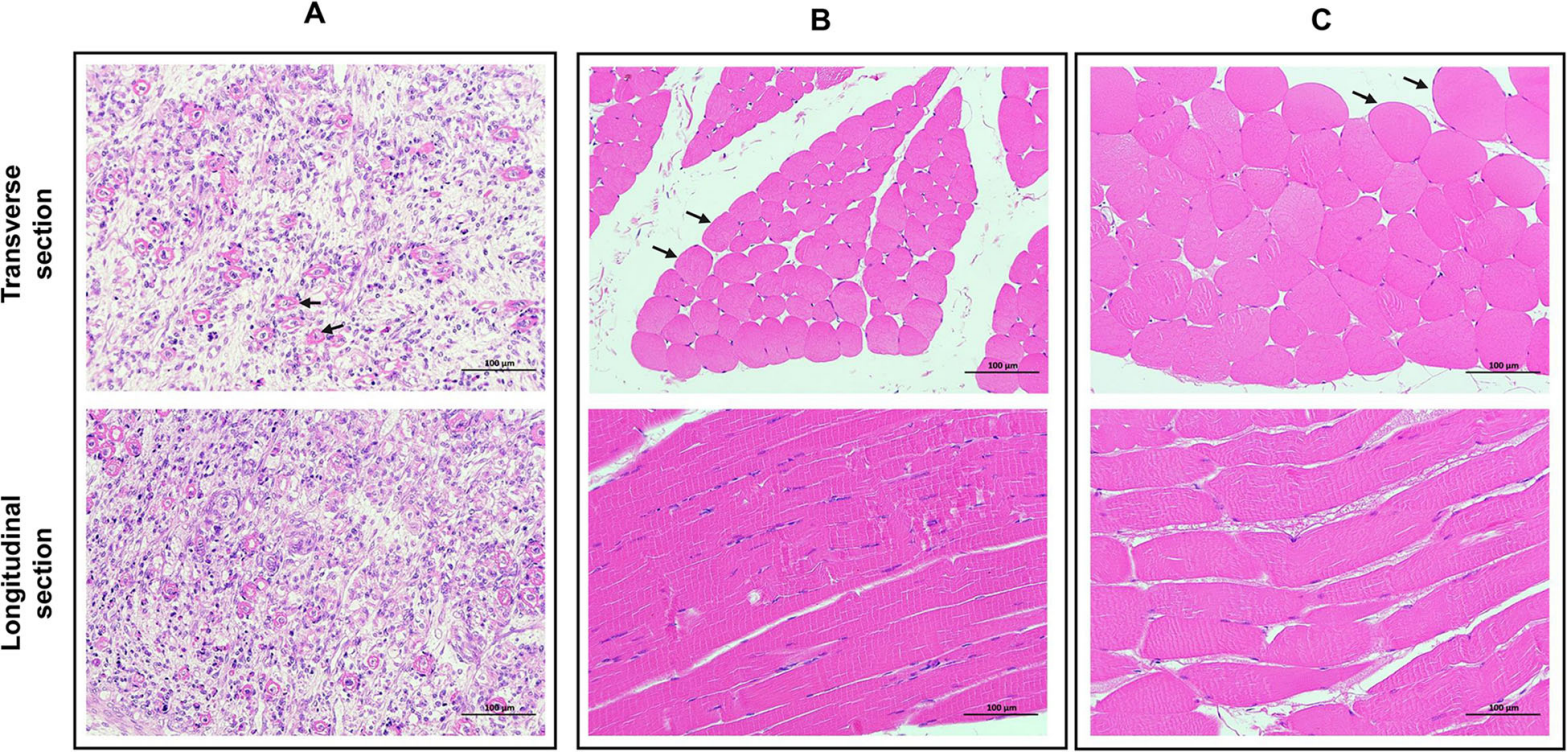
Paraffin section of skeletal muscle of New Zealand rabbit fetus, child and adult. (**A**-**C**) Transverse (top) and longitudinal (bottom) sections of rabbit skeletal muscle during fetus (**A**), child (**B**) and adult (**C**) stages under 10× eyepiece and 20× objective lens. Arrows represent skeletal muscle cells/fibers

To better observe the changes in the characteristics of rabbits’ hind legs skeletal muscles, we generated paraffin sections of the hind legs’ muscles in three developmental stages. From the morphological point of per field, primary muscle fibers with empty tubular shape were mainly observed during the fetus stage, and the number was small (Fig. 1A). In the child stage, complete secondary muscle fibers were formed in the hind legs (Fig. 1B). And in adulthood, the area and diameter of individual muscle fibers increase significantly **(***P* < 0.01, Fig. 1C) At the same time, the number, diameter, area, and density of rabbit muscle fibers in a single field of view were also measured. The results showed that these four characteristics were extremely significantly different in the three developmental stages (*P* < 0.01, Table 1).

**Table 1:** Developmental Characteristic Parameters of Muscle Fibers

Stage	N	Number of muscle fibers per view	Diameter of muscle fibers per view (µm)	Area of muscle fibers per view (µm^2^)	Density of muscle fibers per view
**Fetus**	30	30.1 ± 4.58 ^C^	12.99 ± 0.28 ^C^	453.27 ± 17.72 ^C^	1.11 × 10^− 4 C^
**Child**	30	143.53 ± 12.86 ^A^	30.77 ± 2.72 ^B^	985.72 ± 36.77 ^B^	5.31 × 10^− 4 A^
**Adult**	30	63.60 ± 8.58 ^B^	57.54 ± 1.57 ^A^	3229.16 ± 96.08^ A^	2.36 × 10^− 4 B^

N is the number of views. The different letters in each column indicate significant differences between developmental stages (*P* < 0.01)

### Identification and characterization of circRNAs

Nine libraries of fetuses, child and adult rabbits’ skeletal muscle were constructed. All libraries obtained raw data with an average of 81.03 to 107.01 million. Under strict filtering conditions, libraries average contained 91.68 million effective clean reads, accounting for approximately 99.03 % of the raw reads. And the ratio of effective clean reads in all libraries mapped to the genome was greater than 86.09 %. Furthermore, the Q20 > 97.95 % and Q30 > 93.92 % of all libraries (Addition file 1). For the annotated transcripts, 12,536 protein coding genes were identified (Addition file 2). Besides, find_circ and CIRI2 identified 16,943 candidate circRNAs, derived from 4,115 loci (Addition file 2).

**Fig. 2:**
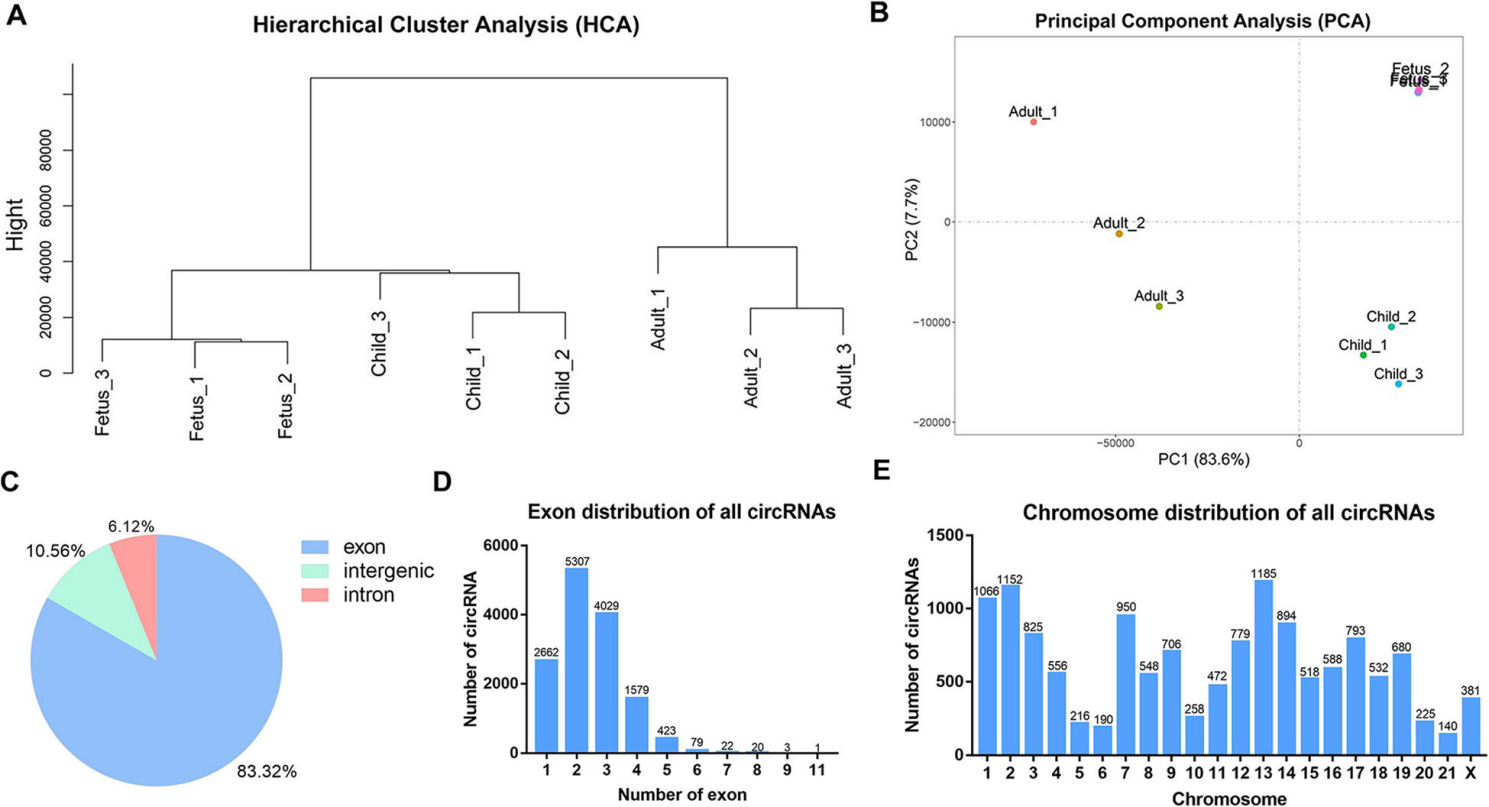
Characterization of candidate circRNAs in rabbit skeletal muscle. (**A**-**B**) Hierarchical cluster analysis (HCA) and principal component analysis (PCA) of circRNAs in 9 rabbit skeletal muscle samples. (**C**) Types of all circRNAs. (**D**) Exon distribution of all circRNAs. (E) Chromosomes distribution of all circRNAs

Hierarchical cluster analysis (HCA) and principal component analysis (PCA) of circRNAs showed that 3 samples at each stage clustered together (Fig. 2 A and B). Then, we analyzed the exons and chromosome distribution of circRNAs. All candidate circRNAs were found to be mainly spliced by exons (83.32 %), introns (6.12 %) and intergenic regions (10.56 %) (Fig. 2C). Among the 83.32 % (14,117) circRNAs, only one has 11 exons. Besides, circRNAs containing 1 ~ 4 exons accounted for 96.17 % (13,577), of which circRNAs containing 2 exons accounted for the highest proportion (37.59 %, 5,307) (Fig. 2D). Additionally, 13,273 circRNAs were unevenly transcribed from the 21 autosome pairs in the rabbit, 381 were derived from the X chromosome. Chromosomes 1, 2, and 13 produced more circRNAs (> 1,000) than any others (Fig. 2E). These results confirmed the reliability of the data and identified the characterization of circRNAs.

### Analysis of differentially expressed circRNAs (DE-circRNAs) and protein coding genes (DEGs)

**Fig. 3:**
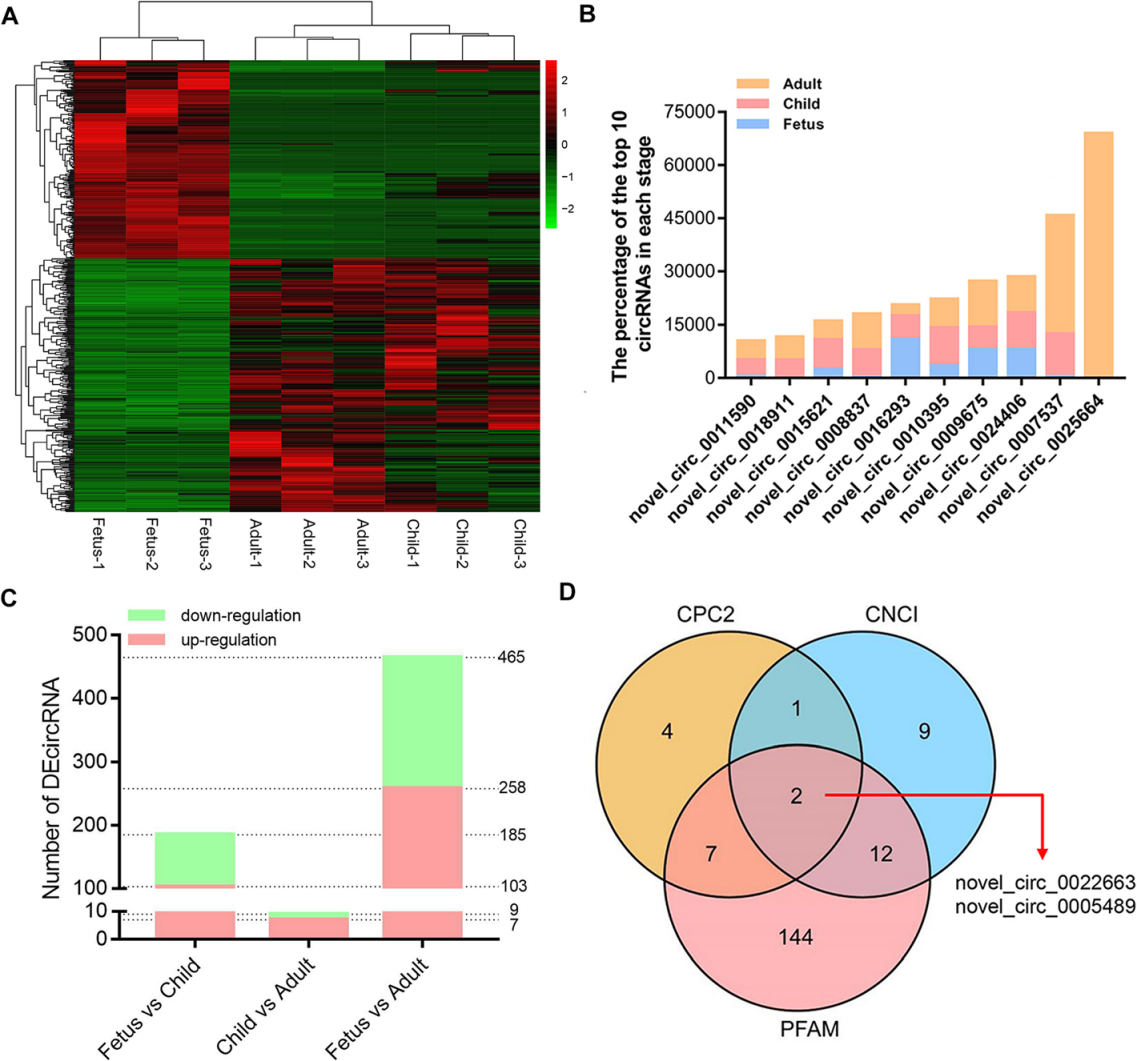
Dynamic changes of DE-circRNAs. (**A**) Hierarchical clustering heat map of all DE-circRNAs. (**B**) DE-circRNAs of fetus vs. child, child vs. adult and fetus vs. adult groups. Red and green represent up-regulated and down-regulated of DE-circRNAs respectively. (**C**) The top 10 circRNAs in expression. Blue, pink and orange represent the expression levels in the fetus, child and adult, respectively. (**D**) Venn diagram of DE-circRNAs with coding potential

To understand the dynamic changes of circRNAs and protein coding genes, the differential expression analyses of circRNAs of rabbits skeletal muscle at the fetus, child and adult stages were performed. Additionally, to filter out false-positive circRNAs, the transcripts expressed in at least three libraries were retained. A total of 481 circRNAs were identified in three groups. The heatmap showed their dynamic changes in all samples (Fig. 3A, Addition file 3). Among them, 185 (103 up- and 82 down-regulation), 9 (7 up- and 2 down-regulation) and 465 (258 up- and 207 down-regulation) DE-circRNA were found in the fetus vs. child, child vs. adult and fetus vs. adult groups, respectively (Fig. 3B, Addition file 3). Next, the circRNAs with the highest expression were focused. Interestingly, novel_circ_0025664, which had the highest expression, was only expressed in adulthood (Fig. 3C). Furthermore, novel_circ_0022663 and novel_circ_0005489 that might have coding ability were identified through Coding Potential Calculator 2 (CPC2), Coding-Non-Coding Index (CNCI) and pfam_scan (PFAM) (Fig. 3D, Addition file 4).

Moreover, protein coding genes were also analyzed. A total of 5,658 DEGs were identified in the three groups (Addition file 3,Addition figure 1 A). There 4,577 DEGs (2,236 up-regulation and 2,341 down-regulation) were found in fetus vs. child group, while 729 DEGs were found in child vs. adult group (351 up- and 378 down-regulation) (Addition figure 1B and C). In the fetus vs. adult group, a total of 4,504 DEGs with 2,152 up-regulated and 2,352 down-regulated were identification (Addition figure 1D).

### The functions of DE-circRNAs in rabbits’ skeletal muscle

**Fig. 4:**
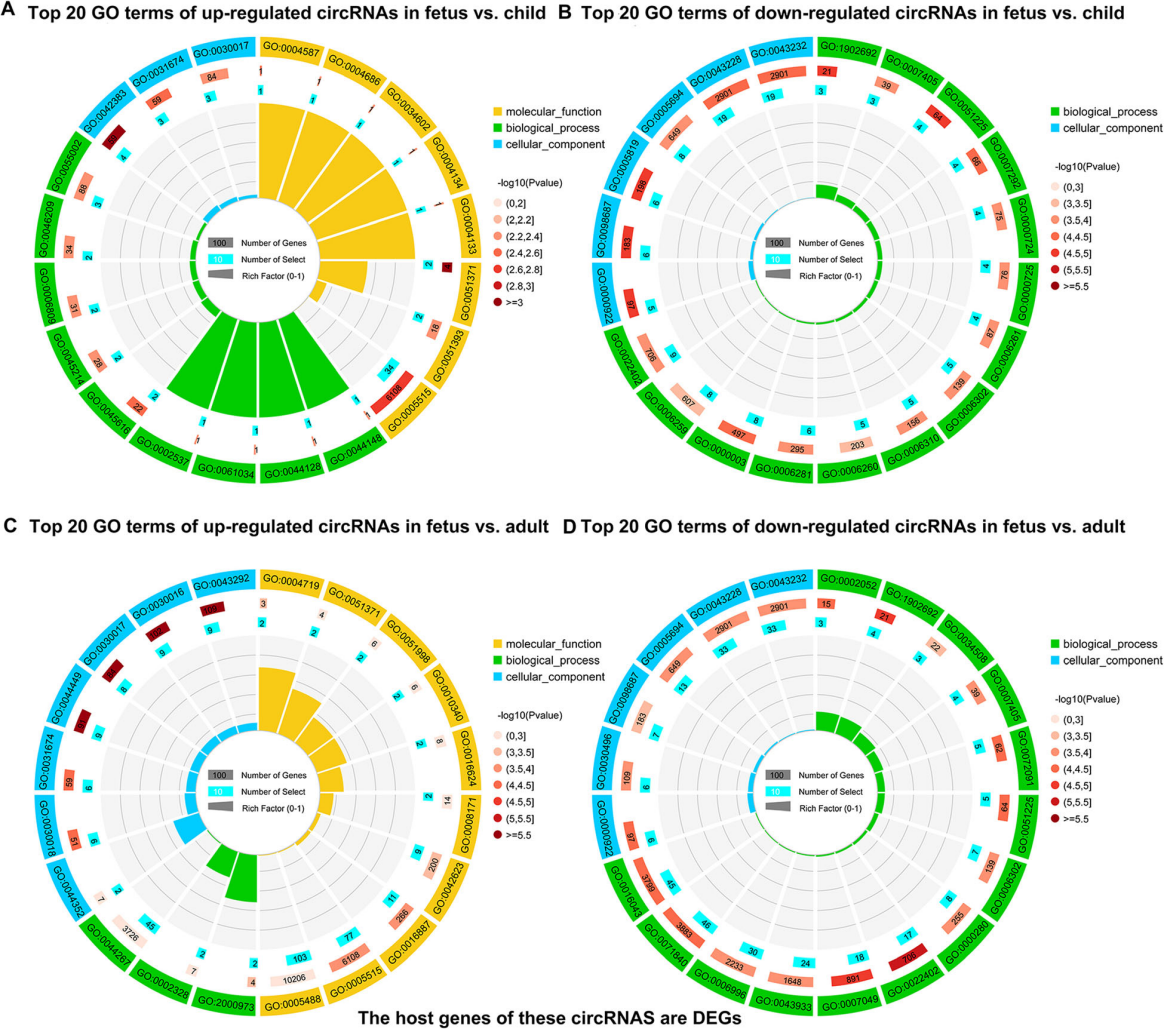
GO enrichment terms of DE-circRNAs (whose host genes are DEGs). (**A**-**D**) The top 20 GO enrichment terms of DE-circRNAs (whose host genes are DEGs) that up-regulated and down-regulated in the fetus vs. child group (**A**-**B**) and fetus vs. adult group (**C**-**D**). There are four circles from outside to inside. The first circle is the classification of GO enrichment terms. Different colors represent different categories. The second circle represents *P*-value and the number of background genes in the category. The more genes, the longer the bar. The smaller the *P*-value, the redder the color. The third circle shows the total number of foreground genes. The fourth circle is the RichFactor value of each category (the number of foreground genes in the category divided by the number of background genes). Each grid of the background auxiliary line represents 0.1

First of all, GO analysis was performed on DE-circRNAs whose host genes were DEGs. The 29 up-regulated DE-circRNAs in the fetus vs. child group had no significantly enriched GO term, but the top 20 terms were related to muscle development, such as “sarcolemma”, “muscle alpha-actinin binding” and “striated muscle cell development”, etc. (Fig. 4A, Addition file 5). The 53 down-regulated DE-circRNAs in this group were associated with cell proliferation, such as “chromosomal region”, “double-strand break repair”, “DNA recombination” and others (Fig. 4B, Addition file 5). Besides, GO analysis showed that the 150 up-regulated DE-circRNAs in the fetus vs. adult group were enriched in 4 terms that related to the formation of skeletal muscle, including “contractile fiber part”, “myofibril”, “contractile fiber” and “sarcomere” (Fig. 4C, Addition file 5). The functions of 125 down-regulated DE-cricRNAs in this group were associated with cell proliferation (Fig. 4D, Addition file 5).

**Fig. 5:**
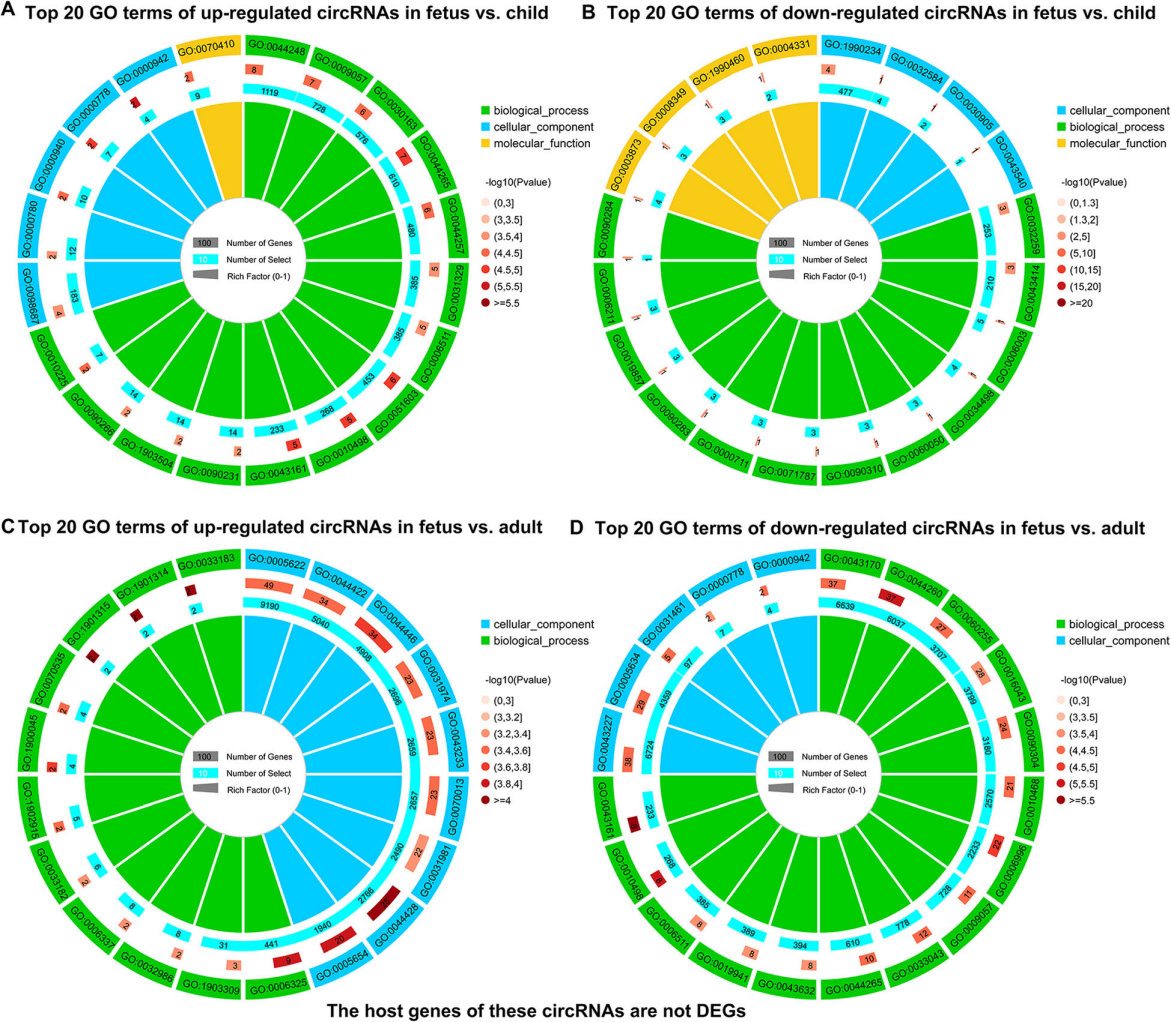
GO enrichment terms of DE-circRNAs (whose host genes are non-DEGs). (**A**-**D**) Top 20 GO enriched terms of host genes of uniquely expressed DE-circRNAs (whose host genes are non-DEGs) that up-regulated and down-regulated in the fetus vs. child group (**A**-**B**) and fetus vs. adult group (**C**-**D**). Detail descriptions are shown in legend of Fig. 4

Meanwhile, in order to reduce background noise, we conducted GO analysis of DE-circRNAs, where the host genes were non-DEGs. The top 20 GO terms of 40 up-regulated DE-circRNAs in the fetus vs. child group mainly involved in enzyme activity, methylation, and glycosylation (Fig. 5A, Addition file 6). The 29 down-regulated DE-circRNAs in this group were mainly involved in mitosis and catabolism (Fig. 5B, Addition file 6). Additionally, 108 up-regulated DE-circRNAs in the fetus vs. adult group were related to the regulation of histone ubiquitination, chromatin, and organelles (Fig. 5C, Addition file 6). The 82 down-regulated DE-circRNAs were related to the catabolism process (Fig. 5D, Addition file 6).

### Rabbit DE-circRNA with potential as miRNA sponge

**Fig. 6:**
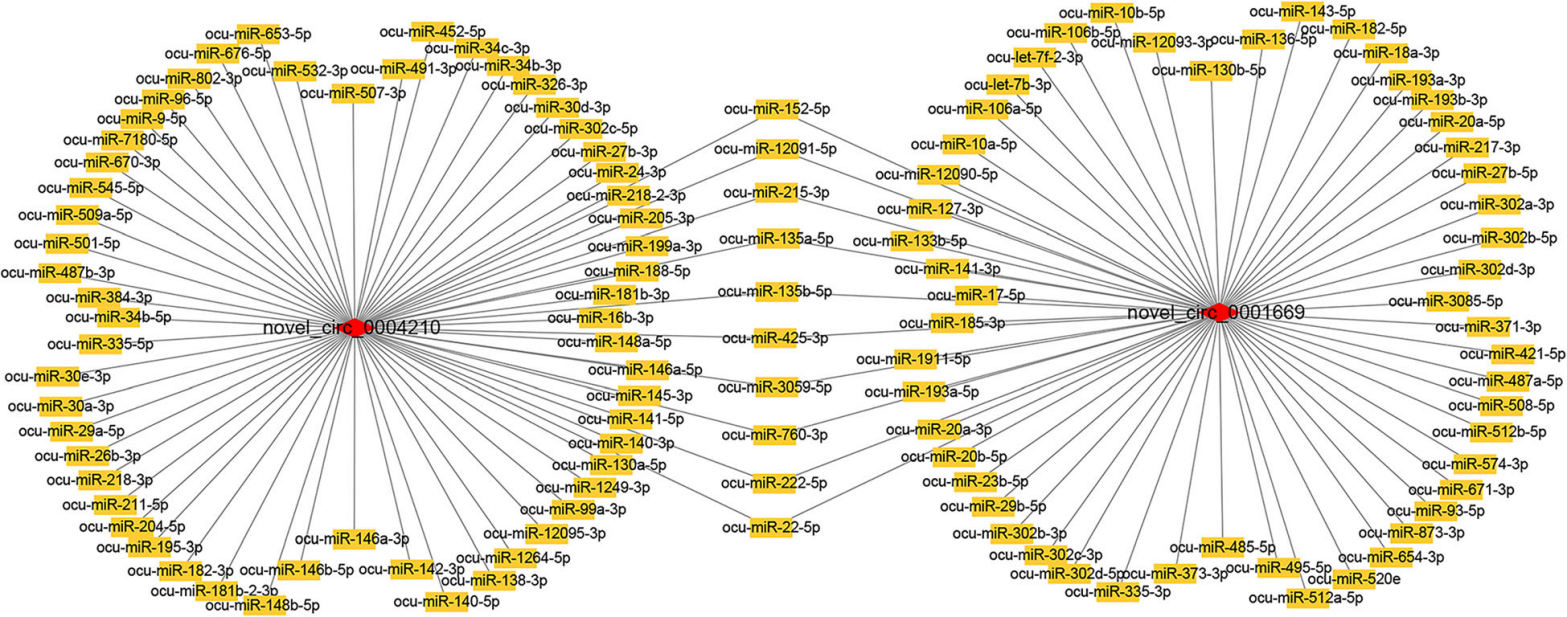
The connection network of novel_circ_0004210 and novel_circ_0001669 with miRNAs respectively The orange box represents miRNA; the red diamond represents DE-circRNA

So as to explore whether the rabbit circRNAs act as miRNA sponges, miRanda was used to predict the miRNA binding sites of DE-circRNAs. A total of 577 miRNAs’ binding sites were targeted by all DE-circRNAs (Addition file 7). Only 1 DE-circRNA had only 1 miRNA binding site, the other DE-circRNAs have more than four binding sites. Besides, the number of miRNA binding sites was unevenly distributed. The data showed that there were 84, 250, 114 and 32 DE-circRNAs with 4–10, 11–20, 21–30 and > 30 miRNA binding sites, respectively. In particular, novel_circ_0004210 and novel_circ_0001669 had 67 and 62 targeted miRNAs, respectively. These two DE-circRNA also shared 10 targeted miRNAs **(**Fig. 6**)**. Moreover, they had the most degrees, so they were selected as the circRNAs with the most potential as miRNA sponges in the skeletal muscle of rabbit hind legs.

### Verification of the expression changes of DE-circRNAs

**Fig. 7:**
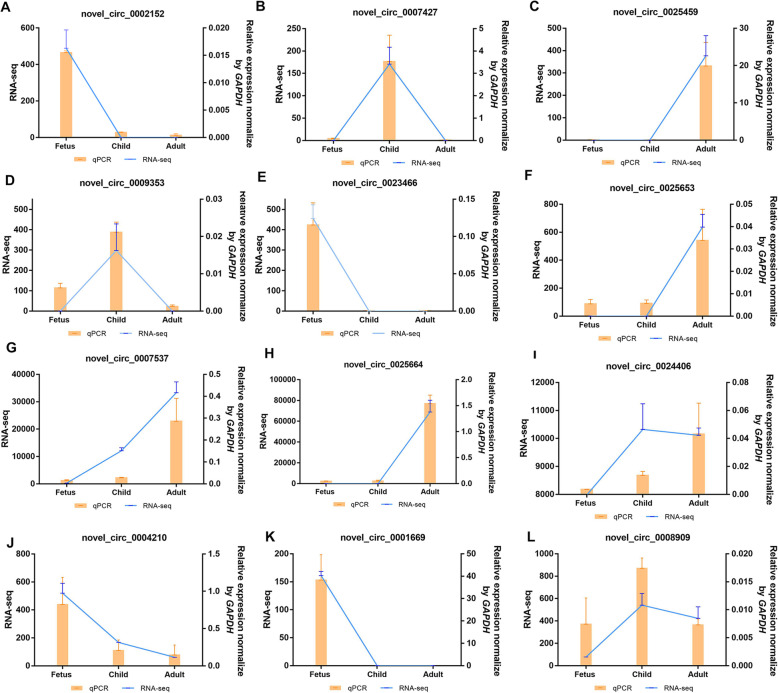
QPCR verification of DE-circRNA. (**A**-**L**) qPCR (Bar chart, orange) and RNA-seq expression (Line chart, blue) validation of twelve rabbit skeletal muscle DE-circRNAs. The circRNAs expression levels were normalized to the *GAPDH*

To prove the circular structure of DE-circRNA, we selected 12 candidates for verification, including novel_circ_0024406, novel_circ_0007537, novel_circ_0025664, novel_circ_0023466, novel_circ_0002152, novel_circ_0007427, novel_circ_0009353, novel_circ_0025459, novel_circ_0025653, novel_circ_0001669, novel_circ_0004210. Subsequently, qPCR verified that the expression profiles of these 11 DE-circRNAs were consistent with the RNA-seq data (Fig. 7). In addition, we designed convergent primers and divergent primers to amplify gDNA and RNase R-treated cDNA, respectively (Additional figure 2). No bands were obtained in all gDNA amplified by divergent primers and cDNA amplified by convergent primers. The gDNA amplified by the convergent primers and the cDNA amplified by the divergent primers showed bands at the corresponding positions (Additional figure 3). These results verified the reliability of the data.

## Discussion

Skeletal muscle development is a dynamic developmental process. It gradually differentiates from mesoderm-derived cells into myoblasts, merges into myotubes, and matures to form muscle fibers [11]. At present, skeletal muscle morphology has been widely measured in pig [12, 13], chicken [14] and sheep [15], while it is rarely studied in rabbit. The researches on rabbit skeletal muscle structure were mainly related to jaw muscle. The jaw muscle fiber diameters of Japanese white rabbits at 4, 9, 12, 18, and 33 weeks were approximately 25, 36, 38, 46, and 52 μm, respectively [16]. Compared with the data in this study, rabbit jaw muscle fibers at 4 weeks were thinner than leg muscle fibers at 2 weeks. But at 18 weeks, the diameter of jaw muscle fibers was larger than that of leg muscles. In addition, it was found that the leg muscles of fetuses’ rabbits were not fully developed at 2 weeks. There were only a few primary muscle cells. When in childhood and adulthood, secondary muscle fibers have been completely formed, and the diameter of muscle fibers has increased significantly. The exploration of skeletal muscle structure revealed the changes of leg muscle fibers in the fetus, child and adult stages, and provided a theoretical basis for a better understanding of rabbit skeletal muscle development.

The growth of skeletal muscle at postnatal stage is mainly achieved by increasing the length and circumference of muscle fibers, rather than by increasing the number of muscle fibers [17–19]. Therefore, the skeletal muscle development regulation molecules in the fetus, child and adult rabbits are the factors that should be considered for the future development of commercial meat production. As a new type of RNAs, circRNA has the characteristics of high stability, evolutionary species conservation and tissue-specific expression patterns. They also participates in the regulation of various biological processes [20]. However, the changes in circRNA expression of rabbits before and after birth are still unclear. Hence, we collected New Zealand rabbit hind legs of fetus, child and adult stage for RNA-seq to explore circRNAs that might have potential regulatory effects on skeletal muscle development. The HCA and PCA analysis showed that the samples at each stage had good repeatability, and the qPCR and PCR results verified the reliability of the data.

A total of 481 DE-cricRNAs were identified. Among them, the top three expression circRNAs were novel_circ_0025664, novel_circ_0007537 and novel_circ_0024406. Among them, the host gene of novel_circ_0025664 was *HCCS*, which coordinated the protective mechanism of the myocardium [21]. Next, host genes of up-regulated and down-regulated DE-circRNAs were analyzed by GO. The GO terms of up-regulated DE-circRNAs (whose host genes were DEGs) in the fetus vs. child group were enriched in muscle development-related functions. For example, the host gene of novel_circ_0008909 is myopalladin (MYPN), which promotes muscle growth through modulation of the serum response factor pathway [22]. The down-regulated circRNAs in this group comprised novel_circ_0018533 whose host gene was zeste homolog 2 (EZH2). *EZH2* maintains a key phase of muscle satellite cell expansion [23]. The host genes of up-regulated DE-circRNAs in the fetus vs. adult group are involved in functions of muscle structure. For instance, myotilin (MYOT), the host gene of novel_circ_0015199, plays a significant role in sarcomere assembly by acting together with α-actinin and filamin C to cross link actin into tightly packed bundles [24]. The host genes of up-regulated DE-circRNAs in this group were related to cell proliferation. *BIRC6*, the host gene of novel_circ_0013522, promotes the proliferation of hepatocytes [25].

Then, the functions of circRNAs whose host genes were non-DEGs were displayed. In the fetus vs. child group, the host genes of 40 up-regulated DE-circRNAs in the fetus vs. child group mainly involved in enzyme activity, methylation, and glycosylation. For example, PFKFB1, the host gene of novel_circ_0026312, has the highest kinase activity to shunt glucose toward glycolysis, to satisfy part of the bioenergetics demand and redox homeostasis requirements [26]. The host genes of 29 down-regulated DE-circRNAs in this group were mainly involved in mitosis and catabolism. Such as, the host genes of novel_circ_0020644 and novel_circ_0001112 were NDC80 and CCNB1, their function is regulation of mitotic spindle checkpoint [27, 28]. Furthermore, host genes of up-regulated DE-circRNAs in the fetus vs. adult group were related to the regulation of histone ubiquitination, chromatin, and organelles. For example, TET1 (host gene of novel_circ_0008893), in the function of negative regulation of chromatin modification, interacts with DNMT3A to affect histone modification, thereby regulating gene expression [29]. The host genes of down-regulated DE-circRNAs were related to the catabolism processes, which induced signaling pathways that regulate the process of muscle loss [30]. These results provide researchers with directions for exploration, the downstream functional are needed to clarify how and whether circulating RNA acts as a center that affects protein expression.

In recent years, the ability of circRNAs as miRNA sponges or coding peptides to participate in biological processes has received increasing attention from researchers [6, 31]. So, the ability of DE-circRNAs as miRNA sponges and their coding potential were analyzed. Only novel_circ_0022663 and novel_circ_0005489 were identified as having coding potential in the three-prediction software. And the IRSE score of them was > 0.7. The host gene *SLC46A3* of novel_circ_0022663, which encoded the lactose membrane protein SLC46A3 as an important transporter [32]. Subsequently, the miRNA binding sites of DE-circRNAs were predicted. Novel_circ_0004210 and novel_circ_0001669 were candidates for the most potential as miRNA sponges in the skeletal muscles of rabbit hind legs. For example, the miR-135b-5p shared by them has been confirmed to promote muscle proliferation by targeting MEF2C [33]. The circRNA coding ability and the interaction between circRNA and miRNA are complex processes, so these predicted results provide ideas for future research.

## Conclusions

In this study, DE-circRNAs expression profiles of fetus, child and adult New Zealand rabbits’ leg muscles were analyzed. Moreover, circRNAs with potential as miRNA sponge ability and coding ability were screened out. In the meantime, the potential functions of circRNA at various stages were also predicted. These results provided a better understanding of the role of circRNAs in skeletal muscle development and a new insight into molecular breeding of meat rabbits.

## Materials and methods

### Sample Collection

New Zealand rabbits in the study were purchased from the animal room of Anhui Medical University (Hefei, China), including child stage rabbits at 6-week-old (0.86 ± 0.083 kg) and adult female rabbits with 2 weeks gestation at 6-month-old (4.37 ± 0.033 kg). When sampling, muscle anesthesia was performed with Jingsongling (Shandong Zibo Veterinary Medicine Co., Ltd., Shandong, China) at 2 mg kg^− 1^ before cesarean section. The fully anesthetized rabbits were euthanized by injecting air into the ear vein. The fetuses were taken out from the uterus of female rabbits by cesarean section. Only 1 fetus (each animal represents 1 repetition) was selected for each female rabbit, that was, there were 3 biological repeats in each stage (9.14 ± 0. 33 g). The left hind leg skeletal muscles were taken as samples. All samples were rinsed 3 times with PBS containing penicillin and streptomycin. Then, the samples were divided into 2 groups. One group was immediately frozen in liquid nitrogen until RNA extraction was needed, the other was sectioned into muscle blocks and stored in muscle-specific fixation solution (Servicebio, Wuhan, China) to generate paraffin sections.

### Paraffin section preparation and analysis

After 24 h of stored with skeletal muscle-specific fixative, the samples were trimmed and placed in dehydration boxes. The dehydration boxes were put into the hanging basket in the dehydration machine (JJ-12 J, Wuhan Junjie Electronics Co., Ltd., Wuhan, China) for dehydration with gradient alcohol. Next, we embedded the wax-soaked tissues in an embedding machine (JB-P5, Wuhan Junjie Electronics Co., Ltd., Wuhan, China). The embedded wax blocks were trimmed and placed in a microtome for 4 μm continuous sectioning. The slices were flattened on warm water at 40 °C and dried in an oven at 60 °C. All paraffin sections were deparaffinized, stained with hematoxylin and eosin. Images were collected using Image Pro® 6.0 under 10× eyepiece and 20× objective lens. There were 3 biological replicates in each stage. For each slice, the number, diameter, and area of muscle fibers in 10 non-overlapping and non-lost visual fields were measured through image J. The density of muscle fibers in each field of view was the area in each field divided by the field of view area (270,063.62 µm^2^). All the data do not conform to the normal distribution, so the Tamhane T2 method of ANOVA in SPSS 19.0 was used for analysis.

### RNA sample detection and library construction

RNA was extracted with an animal total RNA isolation kit (Foregene co., ltd., Chengdu, China). The degradation and contamination of RNA were detected with 1 % agarose gel. RNA purity and integrity depend on the spectrophotometer (IMPLEN, CA, USA) and the RNA Nano 6000 analysis kit of the Bioanalyzer 2100 system (Agilent Technologies, CA, USA), respectively. RNA with an OD260/D280 absorbance of between 1.8 and 2.0 was used for further experiments.

A total of 5 µg RNA extracted from each sample was used as input material for sample preparation. Total RNA removed ribosomal RNA and linear RNA to construct chain-specific libraries. Firstly, ribosomal RNA and rRNA free were removed by Epicenter Ribozero™ rRNA Removal Kit (Epicentre, Madison, WI) and ethanol precipitation respectively. Subsequently, the linear RNA was digested with 3 U of RNase R (Epicentre, Madison, WI) per µg of RNA. The RNA-seq libraries were generated by NEBNext® Ultra™ Directional RNA Library Prep Kit for Illumina® (NEB, MA USA) following the manufacturer’s recommendations. The library fragments were purified with the AMPure XP system (Beckman Coulter, Beverly, USA) into cDNA fragments with a preferred length of 250 ~ 300 bp. Next, 3 µL of USER enzyme (NEB, MA, USA) was used with the size-selected. Then, PCR was performed after using 3 µL of USER enzyme (NEB, MA, USA) for size-selected and adaptor-ligated cDNA at 37 °C for 15 min followed by 95 °C for 5 min. Finally, products were purified (AMPure XP system) and library quality was assessed on the Agilent Bioanalyzer 2100 system.

### Clustering and quality control of RNA-seq libraries

The clustering of the index-coded samples was performed on a cBot Cluster Generation System by TruSeq PE Cluster Kit v3-cBot-HS (Illumina). Then, the libraries were sequenced with Illumina PE150 (Pair end 150) according to the effective concentration. Four fluorescently labeled dNTPs, DNA polymerase, and adaptor primers were added to the sequencing flow cell for amplification. The Illumina platform captured the fluorescent signal and converted the light signal into a sequencing peak to obtain sequence information.

Clean reads were filtered from raw reads, which were processed through in-house Perl scripts (ng-qc, parameter: -L 20, -p 0.5). The filter conditions included removing reads that low-quality, adaptor-containing, and ploy N-containing. Clean reads were the basis for all downstream data analyses. Q20, Q30 and GC contents were also calculated.

### Identification of circRNAs and protein coding genes

Reference genome and gene models’ FA and GTF files of rabbit (*Oryctolagus cuniculus*) were downloaded directly from the web of ensemble (Genome assembly: OryCun2.0 GCA_000003625.1). Index of the reference genome was built using bowtie2 (criteria: --end-to-end --sensitive --phred33; --no-mixed -X 600) and paired-end clean reads were aligned by Hisat2 (criteria: --no-unal -t; --phred33; --rna-strandness RF --dta-cufflinks; --un-conc-gz) [34]. Subsequently, reads alignment results were transferred to the program StringTie (criteria: --rf -e) for genes assembly. Finally, the circRNAs were detected and identified using find_circ (criteria: default parameters) [35] and CIRI2 (criteria: -P -T 4) [36].

### Differential expression analysis of circRNAs and protein coding genes

The raw counts of the obtained circRNAs were normalized by TPM, which represented the expression level of the circRNAs. Quantification of the genes was performed using StringTie and fragments Per Kilobase of transcript sequence per Millions base pairs sequenced (FPKM) was obtained.

The differential analyses of circRNAs and protein coding genes were performed based on the negative binomial distribution of DESeq2 (R-3.1.2; criteria: --*p*adj 0.05). The input data of circRNA differential expression analysis was read counts. The resulting *P*-values were adjusted by the Benjamini and Hochberg’s approach for controlling the false discovery rate. CircRNAs and protein coding genes with an adjusted *P* (*P*-adj) < 0.05 were assigned as DE-circRNAs and DEGs.

### Function analysis of DE-circRNAs

In the functional enrichment analysis, circRNAs whose host genes were DEG and non-DEG were respectively mapped to each entry in the GO database. Second, according to the number of genes in each term, a hypergeometric test was applied to find out which genes were significantly enriched GO term compared to the entire genome background. For host genes, GO analysis was performed using the GOseq R package [37]. Gene functions were classified into three subgroups namely BP, CC and MF. And GO terms that met *P*-adj < 0.05 were defined as significantly enriched.

### miRNA binding site and coding potential analysis

CircRNA inhibits the function of miRNA in the form of a sponge. Additionally, recent studies have proved that circRNA with ribosome entry site (IRES) and open reading frame (ORF) may be translated, and the translation product plays an important role in biological processes [38]. Therefore, the miRNA binding site and coding potential analysis of the identified circRNAs are helpful to further study the function of circRNA. miRNA binding sites in exons of circRNAs loci were identified using miRanda (miRanda-3.3a, criteria: -sc 140 -en -10; -scale 4 -strict). IRES element prediction of circRNA predicted by IRES Finder. And then the coding potential predicted by CPC2 (V3.2.0, criteria: default parameters) [39], CNCI (criteria: -m ve -p 1)[40] and PFAM (v1.3, criteria:-pfamB) [41]. The collection was circRNAs with coding potential.

### Quantitative PCR

The RNAs treated with RNase R were reverse transcribed into cDNAs, which were used as templates for qPCR. qPCR was performed by 2×Q3 SYBR qPCR Master mix (TOLOBIO, Shanghai, China) and Real-time Thermal Cycler 5100 (Thermo, Shanghai, China). The primer pairs designed by PrimerSelect in DNAstar and synthesized by the TsingKe biological technology company (Addition file 8)**.** The *GAPDH* housekeeping gene was amplified as a control [42]. The circRNAs expression levels were normalized to the reference sequence and calculated as 2^−ΔΔCt^. ANOVA analysis of the normalized data was then conducted using SPSS version 19.0 for Windows [43]. Data were presented as mean ± SE.

## Supplementary Information

**Additional file 1:** **Figure S1:**Dynamic changes of DEGs. (A) Hierarchical clustering heat map of all DEGs. (B-D) Volcano plots of DEGs in fetus vs. child (B), child vs. adult (C) and fetus vs. adult (D) groups.

**Additional file 2:Figure S2: **The primer design of twelve DE-circRNAs.

**Additional file 3:Figure S3: **Agarose gel electrophoresis verified the reliability of DE-circRNAs. (A-L) Agarose gel of gDNA and RNase R-treated cDNA amplified by convergent and divergent primers. C: convergent primers; D: divergent primers; M: maker.

**Additional file 4:** Data overview of all libraries.

**Additional file 5:** Details of all candidate circRNAs and protein coding genes.

**Additional file 6:** *P*-adj of all DE-circRNAs (sheet 1) and DEGs (sheet 2).

**Additional file 7:** Prediction coding ability of DE-circRNAs.

**Additional file 8:** GO analysis of up-and down- regulated DE-circRNAs (whose host genes are DEGs) in the fetus vs. child (sheet 1 and 2) and fetus vs. adult (sheet 3 and 4) groups.

**Additional file 9:** GO analysis of up- and down- regulated DE-circRNAs (whose host genes are non- DEGs) in the fetus vs. child (sheet 1 and 2) and fetus vs. adult (sheet 3 and 4) groups.

**Additional file 10:** miRNAs with DE-circRNA binding sites.

**Additional file 11:** Convergent and divergent primer pairs used for qPCR and PCR amplification.

## Data Availability

The original data files have been uploaded and published to the NCBI SRA database. The accession number is PRJNA681527, the name is “rabbit skeletal muscle of hid leg in fetal, child and adult” (https://www.ncbi.nlm.nih.gov/bioproject/PRJNA681527/).

## Abbreviations

CircRNA: circular RNA
DE-circRNA: differential expression circRNA
DEG: Differential expression protein coding genes
GO: Gene Ontology
HCA: Hierarchical cluster analysis
PCA: Principal component analysis
CPC2: Coding Potential Calculator 2
CNCI: Coding-Non-Coding Index
PFAM: Pfam_scan
