# Supplementary figures and images for: Morphological changes and functional circRNAs screening of rabbit skeletal muscle development
Source: BMC Genomics. 2021 Jun 24;22:469. doi: 10.1186/s12864-021-07706-y (PMC8223307; doi:10.1186/s12864-021-07706-y)

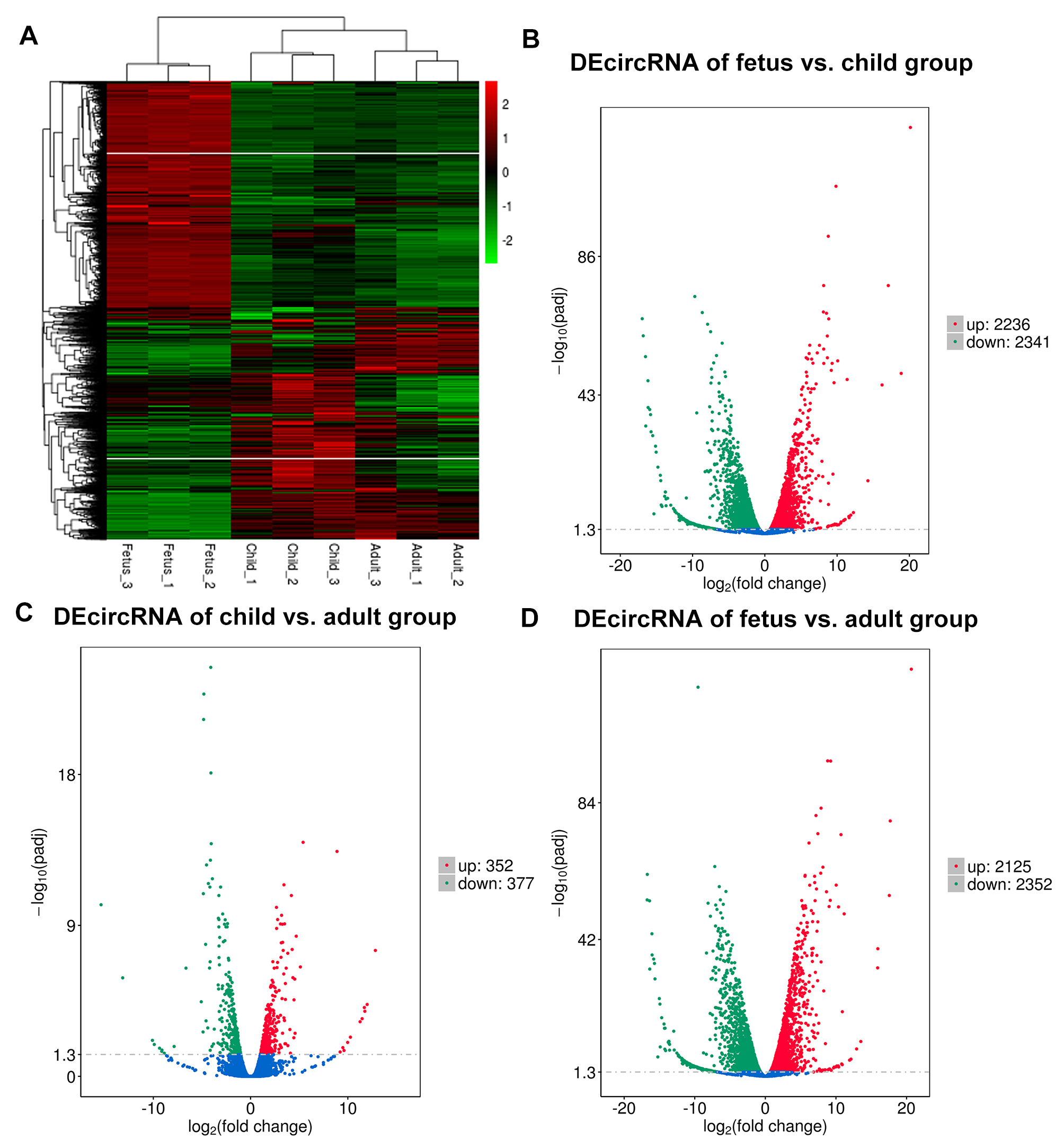

Supplement: Supplementary file 1 — Additional file 1: Figure S1:Dynamic changes of DEGs. (A) Hierarchical clustering heat map of all DEGs. (B-D) Volcano plots of DEGs in fetus vs. child (B), child vs. adult (C) and fetus vs. adult (D) groups. [file 12864_2021_7706_MOESM1_ESM.jpg]

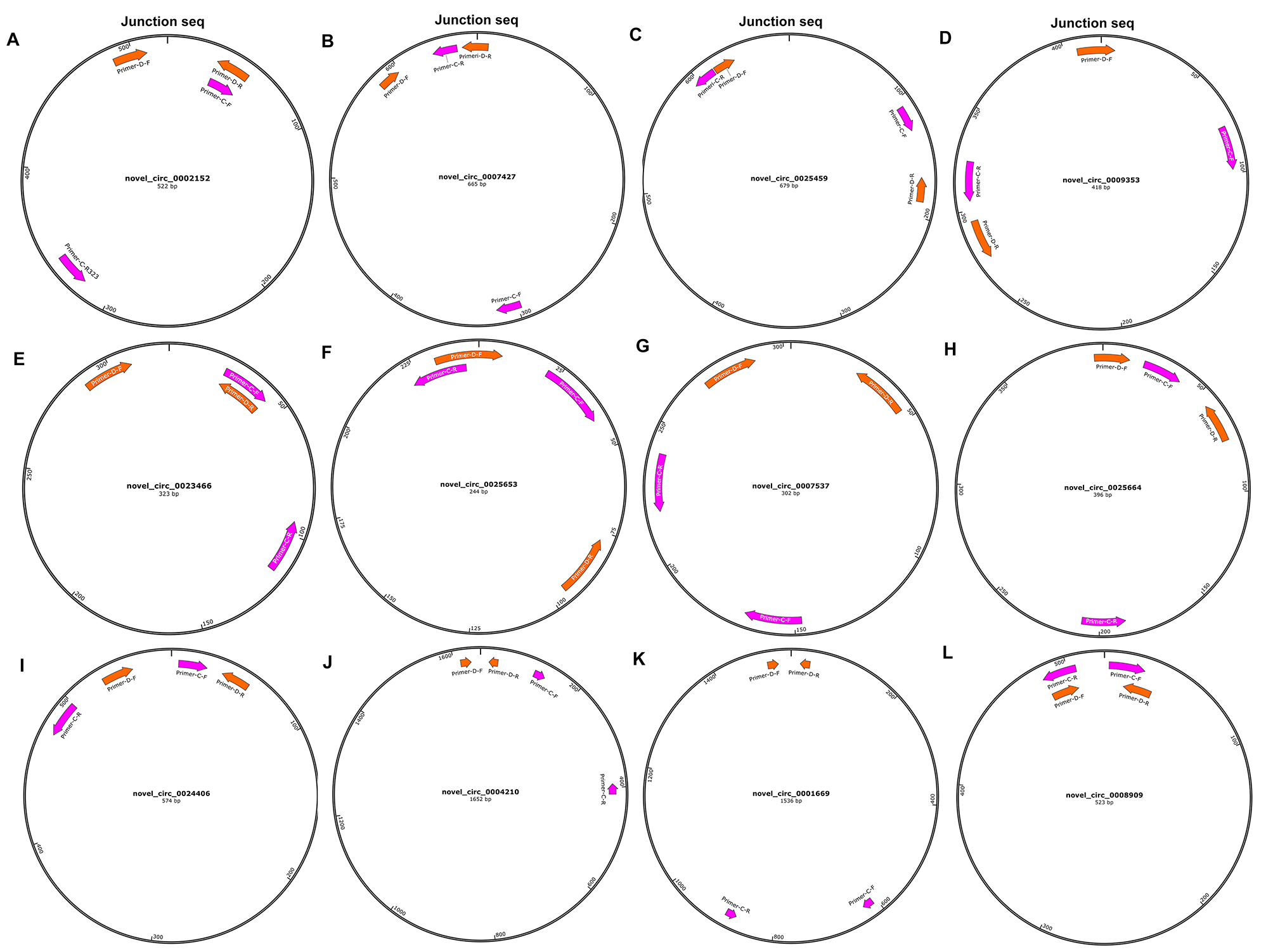

Supplement: Supplementary file 2 — Additional file 2:Figure S2: The primer design of twelve DE-circRNAs. [file 12864_2021_7706_MOESM2_ESM.jpg]

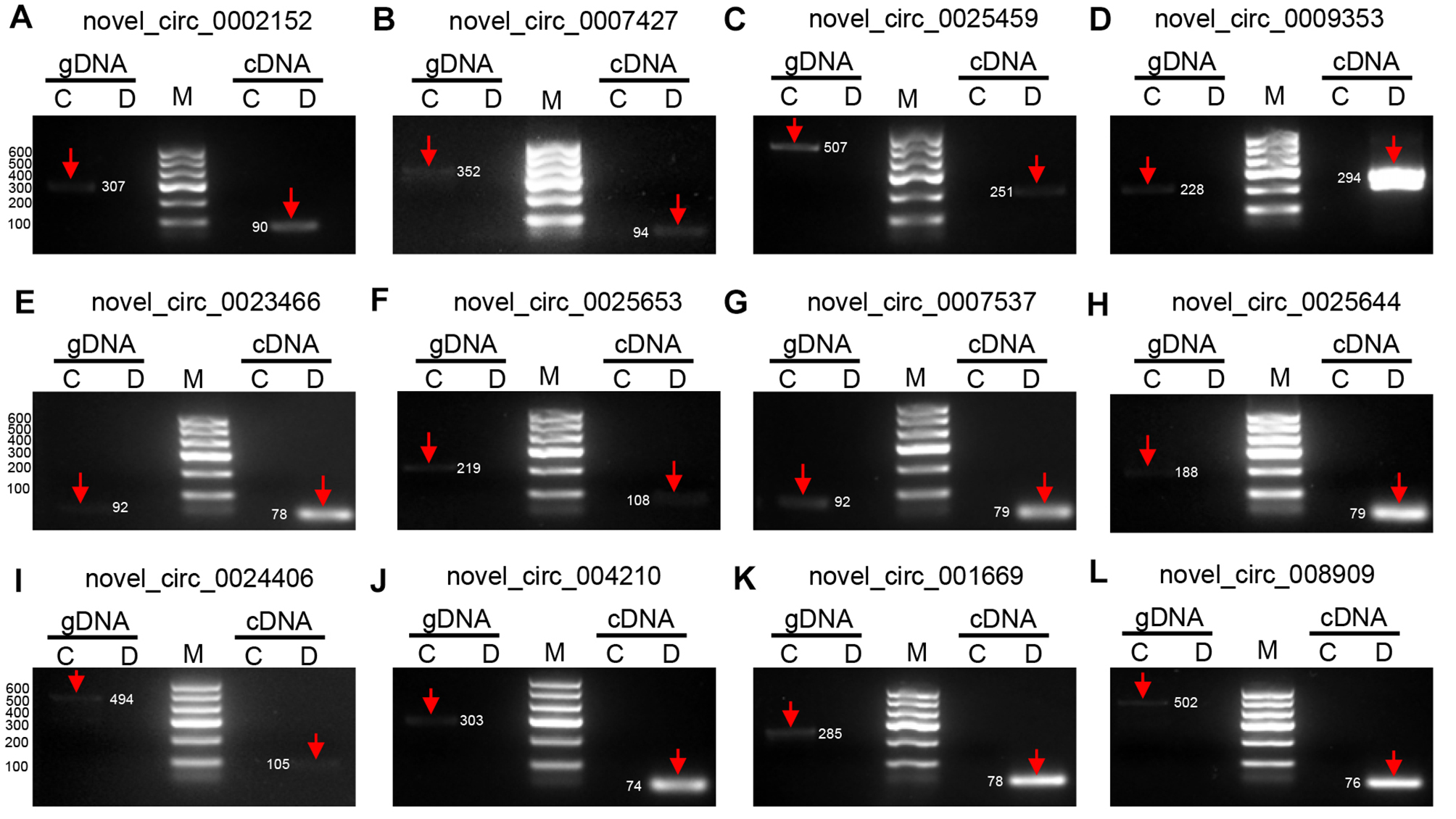

Supplement: Supplementary file 3 — Additional file 3:Figure S3: Agarose gel electrophoresis verified the reliability of DE-circRNAs. (A-L) Agarose gel of gDNA and RNase R-treated cDNA amplified by convergent and divergent primers. C: convergent primers; D: divergent primers; M: maker. [file 12864_2021_7706_MOESM3_ESM.jpg]
